# Lignin-Based Cationic Hydrogels Incorporating MIL-100(Fe) for Combined Adsorption and Photo-Fenton Degradation of Naproxen Sodium

**DOI:** 10.1021/acsaenm.6c00333

**Published:** 2026-06-05

**Authors:** Simone Ranieri, Paola Astolfi, Marco Parlapiano, Massimiliano Sgroi, A. Rabdel Ruiz-Salvador, Menta Ballesteros, Michela Pisani

**Affiliations:** † Department of Science and Engineering of Materials, Environment and Urban Planning, 9294 Marche Polytechnic University, via Brecce Bianche 12, Ancona 60131, Italy; ‡ Department of Physical, Chemical and Natural Systems, 16772 Universidad Pablo de Olavide, Ctra. de Utrera, Km. 1, Seville 41013, Spain; § Department of Molecular Biology and Biochemical Engineering, Experimental Sciences Faculty, Universidad Pablo de Olavide, Ctra. de Utrera Km 1, Seville 41013, Spain; ∥ Center for Nanoscience and Sustainable Technologies (CNATS), Universidad Pablo de Olavide, Ctra. Utrera Km. 1, Seville 41013, Spain

**Keywords:** water treatment, hydrogel, MOF, naproxen, photo-fenton

## Abstract

The occurrence of persistent pharmaceutical residues, such as nonsteroidal anti-inflammatory drugs (NSAIDs), in aquatic environments requires treatment strategies that combine high removal efficiency with operational practicality. Herein, a hybrid lignin-based cationic hydrogel incorporating the iron-based metal-organic framework MIL-100­(Fe) (LS-pAAm-DAC/MIL-100­(Fe)) is reported as a multifunctional material for the integrated adsorption and heterogeneous photo-Fenton degradation of naproxen sodium (NPX-Na) in water. The composite is synthesized *via in situ* radical polymerization, leading to the uniform immobilization of crystalline MIL-100­(Fe) within a sustainable, lignin-derived polymer network. Structural and morphological analyses (XRD, ATR-FTIR, SEM/EDS) confirm the preservation of the MOF structure and its homogeneous dispersion throughout the hydrogel matrix. The composite exhibits rapid NPX-Na uptake and enhanced adsorption capacity (39 mg/g) compared with the pristine hydrogel, attributable to combined electrostatic interactions in the cationic network and additional adsorption sites on MIL-100­(Fe). Under UVA irradiation in the presence of H_2_O_2_, the material promotes complete NPX-Na degradation *via* a heterogeneous photo-Fenton process, with HPLC/MS evidence of progressive transformation into oxidized phthalic acid-type by-products. Importantly, ICP-OES analysis reveals no detectable iron leaching, demonstrating robust immobilization of MIL-100­(Fe) and operational stability. The composite retains substantial adsorption performance over multiple regeneration cycles, highlighting its reusability. Overall, LS-pAAm-DAC/MIL-100­(Fe) represents a recoverable and sustainable platform that integrates capture and oxidative degradation of anionic pharmaceuticals, offering promising prospects for advanced water treatment applications.

## Graphical abstract

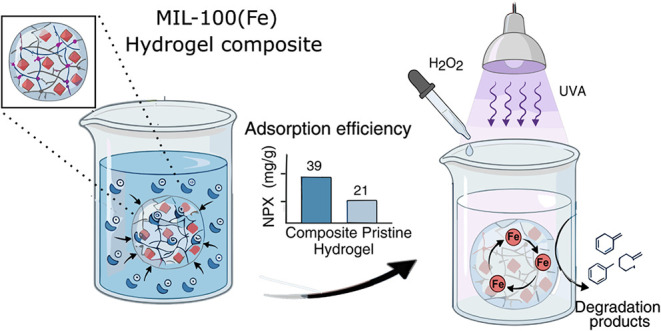

## 1 Introduction

The widespread occurrence of pharmaceutical residues in aquatic ecosystems has emerged as a concerning environmental issue. Among these, nonsteroidal anti-inflammatory drugs (NSAIDs) are of particular concern due to their extensive consumption, partial metabolism, and resistance to conventional wastewater treatments. Naproxen sodium (NPX-Na), a widely used NSAID, is frequently detected in surface and groundwater at concentrations ranging from ng/L to μg/L. Its high environmental persistence, potential ecotoxicity, and inefficient elimination by wastewater treatment plants (WWTPs) have raised significant concern.^1–3^ Conventional methods employed for the removal of NPX-Na and other emerging contaminants, including filtration, coagulation-flocculation/settling, pressure-driven membranes, etc., often suffer from limited efficiency, poor selectivity, or high operational costs.^4,5^ While these technologies are widely implemented at full scale, several critical reviews have highlighted their limited effectiveness toward persistent and hydrophilic pharmaceutical compounds.^6,7^ To overcome these limitations, Advanced Oxidation Processes (AOPs), including Fenton-like and persulfate-assisted systems,^8–10^ have garnered attention for their capacity to generate highly reactive oxygen species (ROS) capable of degrading recalcitrant pollutants. In this context, iron-based remediation technologies have progressively evolved from the use of zero-valent and supported iron adsorbents toward catalytic iron-containing materials capable of activating oxidants such as H_2_O_2_, persulfate, and peroxymonosulfate for enhanced pollutant degradation.^11–13^ More recently, iron-containing metal–organic frameworks (MOFs) have attracted considerable attention because of their tunable porosity, accessibility of catalytic sites, and ability to promote heterogeneous photo-assisted oxidation reactions.^14–16^ Among these, MIL-100­(Fe) stands out as a highly porous iron-based MOF, offering excellent stability, a large surface area, and redox-active Fe­(III) centers capable of generating hydroxyl radicals (•OH) in the presence of H_2_O_2_ and light through a heterogeneous photo-Fenton process.^17^ Moreover, MIL-100­(Fe) possesses intrinsic adsorption capacity due to its open metal sites and large pore volume,^18^ which could also contribute to the removal of contaminants prior to catalytic degradation. Taken together, these properties provide MIL-100­(Fe) with the ability to efficiently adsorb and degrade persistent organic pollutants, such as pesticides, antibiotics, and NSAIDs.^19,20^ Despite these advantages, powdered MIL-100­(Fe) has a major limitation: its small particle size and colloidal stability in aqueous media make post-treatment recovery challenging, often requiring ultracentrifugation or membrane filtration.^21^ Such separation methods are impractical for large-scale applications and limit the direct use of MIL-100­(Fe) in continuous flow systems. Consequently, the development of multifunctional, recyclable materials capable of integrating adsorption, catalytic degradation, and easy recovery is strongly requested.

In this context, hydrogel matrices can serve a dual function, acting both as active adsorbents and as physical supports for MOF immobilization.^22–24^ Due to their three-dimensional hydrophilic networks, adjustable porosity, and facile chemical functionalization, hydrogels have attracted considerable interest as efficient adsorbent materials for water purification applications.^25,26^ In our previous work, we developed a lignin-based hydrogel system comprising sulfonated lignin, acrylamide, and a cationic moiety (DAC), which demonstrated effective adsorption of diclofenac sodium, another anionic NSAID.^27^ This material (LS-pAAm-DAC) was used both in batch and fixed-bed configurations, achieving significant contaminant uptake *via* electrostatic attraction, hydrogen bonding, and π–π interactions. However, adsorption alone cannot fully eliminate the pollutant, and the hydrogel lacked any intrinsic degradative activity. Nevertheless, lignin-based hydrogels represent attractive supports for MOF immobilization, as they provide a sustainable and recoverable scaffold while facilitating catalyst handling, recovery, and recyclability.

In this study, we report the synthesis, characterization, and performance assessment of LS-pAAm-DAC/MIL-100­(Fe), a novel hybrid material composed of a cationic lignin-based hydrogel incorporating MIL-100­(Fe). The composite material was specifically designed to combine the adsorption of NPX-Na with its photocatalytic degradation *via* a heterogeneous photo-Fenton mechanism under UVA irradiation and in the presence of H_2_O_2_. By integrating both adsorption and photodegradation capabilities into a single sustainable material, this work presents a scalable and environmentally friendly approach for the removal of NPX-Na from water, a strategy potentially extendable to other persistent contaminants.

## 2 Experimental Section

### 2.1 Materials

Iron­(II) sulfate heptahydrate (FeSO_4_·7H_2_O), 1,3,5-benzenetricarboxylic acid (trimesic acid, H_3_BTC), sodium naproxen (NPX-Na), sodium lignosulfonate (LS), acrylamide (AAm), *N*,*N*’-methylenebis­(acrylamide) (MBAA), [2-(acryloyloxy)­ethyl]­trimethylammonium chloride (80 wt % solution in water) (DAC), tetramethylethylenediamine (TEMED), potassium persulfate (KPS), hydrogen peroxide (30%) (H_2_O_2_), catalase from bovine liver, sodium hydroxide, and HPLC-grade solvents were purchased from Merck (Germany) and used without further purification. Ultrapure water was obtained from a Younglin Aqua Max Ultra 370 (Germany) and used throughout all the procedures.

### 2.2 Synthesis of MIL-100­(Fe)

MIL-100­(Fe) was synthesized under basic conditions following the procedure reported by Steenhaut et al.,^28^ with slight modifications. First, 1.42 g of H_3_BTC and 0.80 g of sodium hydroxide were dissolved in 125 mL of ultrapure water under ultrasonic agitation until complete dissolution. Separately, 2.25 g of FeSO_4_·7H_2_O was dissolved in 125 mL of ultrapure water. The two solutions were then combined in a 500 mL Erlenmeyer flask and stirred at 500 rpm for 15 h at room temperature. Upon mixing, the solution initially turned dark green, gradually shifting to orange as the oxidation of the iron­(II) species to iron­(III) proceeded and a precipitate was formed. This latter was recovered by centrifugation (10000 rpm, 20 min), washed three times with 50 mL of deionized water and one time with 50 mL of ethanol, and then dried for 5 h at 60 °C in an oven. The sample was further dried under vacuum until a constant weight was achieved.

### 2.3 Preparation of LS-pAAm-DAC/MIL-100­(Fe) Composite

The lignin-based composite hydrogel was synthesized *via* radical polymerization, following a previously reported protocol with slight modifications.^29^ In this procedure, 400 mg of sodium lignosulfonate (LS) was dissolved in 20 mL of ultrapure water under constant stirring at room temperature, along with 1400 mg of AAm and 36 mg of the cross-linker MBAA. The mixture was stirred at 300 rpm for 30 min to ensure complete dissolution. The MIL-100­(Fe) powder, synthesized as described in Section 2.2, was then added (600 mg) directly to the reaction mixture and homogenized by stirring. Subsequently, 770 μL of DAC and 70 μL of TEMED were added to the reaction mixture, and after a short equilibration period, 220 mg of KPS was introduced as the initiator. Gelation occurred shortly thereafter, and the reaction was allowed to proceed at 40 °C for 4 h to ensure complete network formation. The resulting composite material was thoroughly washed with deionized water and acetone to remove unreacted monomers. For the chemical-physical characterization, the samples were frozen at −80 °C for 2 h and then freeze-dried at −40 °C for 40 h using a Superco Engineering Proxima Lyophilizer (Germany) operating in autodrying mode at 0.38 mbar vacuum and then in a secondary drying mode at 0.25 mbar.

### 2.4 Characterization of LS-pAAm-DAC/MIL-100­(Fe) Composite

#### 2.4.1 Attenuated Total Reflection Fourier Transform Infrared (ATR-FTIR) Measurements

ATR-FTIR spectra were collected on a Bruker INVENIO-R spectrometer fitted with a Platinum ATR accessory with a diamond crystal and a Deuterated TriGlycine Sulfate (DTGS) detector (Bruker Optics, Ettlingen, Germany). For each analysis, a few milligrams of sample were deposited directly onto the diamond crystal, and measurements were performed in triplicate at room temperature over the range of 4000–400 cm^–1^ using 128 scans at a spectral resolution of 4 cm^–1^. Background spectra were acquired using the same parameters. The acquired spectra were vector-normalized over the entire spectral range using OPUS 7.5 software (Bruker Optics, Ettlingen, Germany).

#### 2.4.2 Powder X-ray Diffraction (XRD)

Phase identification of MIL-100­(Fe) powder and of the LS-pAAm-DAC/MIL-100­(Fe) composite was carried out by X-ray diffraction using a Bruker D8 Discover diffractometer equipped with a 2D detector at the Integrated Materials Characterization Laboratory (INMALAB), Pablo de Olavide University (Seville, Spain). Data collection was controlled through Diffrac Commander software using Cu K_α_ radiation (λ = 1.5406 Å), an accelerating voltage of 50 kV, and a tube current of 1000 μA. Diffractograms were acquired in continuous mode over 2θ = 2–35°, with a step size of 0.02° and a counting time of 0.3 s per step. The resulting patterns were analyzed using Diffrac Suite software. Prior to analysis, the samples were lightly ground and evenly distributed on a flat sample holder. For the composite material, diffraction patterns were additionally recorded at ten randomly chosen points on the specimen to assess spatial uniformity and confirm the retention of MIL-100­(Fe) within the polymer matrix. The experimental diffractograms were compared with MIL-100­(Fe) reference data.

#### 2.4.3 Scanning Electron Microscopy (SEM) and Energy-Dispersive X-ray Spectroscopy (EDS)

Morphological analysis was carried out on MIL-100­(Fe) powder and MIL-100­(Fe)-loaded freeze-dried composite by SEM (ZEISS SUPRA 40, Oberkochen, Germany), acquiring images at multiple magnifications to evaluate surface features, and the dispersion of MOF particles within the polymeric network. MIL-100­(Fe) powder and MIL-100­(Fe)-loaded freeze-dried composite specimens were mounted on aluminum stubs using carbon adhesive tape and sputter-coated with a thin gold layer to minimize charging. Unless otherwise stated, SEM observations were performed at an accelerating voltage of 25 kV with a working distance of 15 mm. Elemental composition and spatial distribution were assessed on the same specimens by an EDS (Bruker Quantax 200-Z10) microanalysis system. Fe maps were collected to assess MIL-100­(Fe) incorporation and spatial distribution. Point and area spectra were processed, and elemental concentrations were reported as weight percent (wt %).

#### 2.4.4 Swelling and Water Retention Behavior

Swelling and water retention behavior of the LS-pAAm-DAC/MIL-100­(Fe) composite were quantified gravimetrically. For swelling assays, freeze-dried specimens were accurately weighed and immersed in ultrapure water at room temperature until equilibrium. At predetermined intervals, samples were withdrawn, blotted with filter paper to remove superficial liquid, and weighed; measurements continued until no further mass changes were observed. The swelling degree was obtained from eq 1:^27^

1 $$S\left(\%\right)=\frac{M_{t}-M_{0}}{M_{0}}\times 100$$

where *M*_0_ and *M_t_* are the masses of the dry and swollen composite at time *t*, respectively.

Water retention was evaluated by equilibrating the composite samples in ultrapure water and leaving them to dry in open air at room temperature while measuring the mass at set time intervals until no further changes were observed. Residual water content was expressed as water retention (WR%) according to eq 2:

2 $$WR\left(\%\right)=\frac{M_{t}-M_{0}}{M_{e}-M_{0}}\times 100$$

where *M*_0_ is the weight of the dried composite, *M*_e_ and *M*_*t*_ are the weights of the swollen composite at equilibrium time and at time *t*, respectively. Swelling and water retention measurements were performed in triplicate on independent specimens; results are reported as mean ± SD (*n* = 3).

### 2.5 Adsorption and Photodegradation of Naproxen Sodium (NPX-Na)

#### 2.5.1 Adsorption and Desorption of NPX-Na

Batch adsorption tests were performed to evaluate the NPX-Na uptake by LS-pAAm-DAC/MIL-100­(Fe). Composite samples (0.5 g) were immersed in 100 mL of NPX-Na (100 mg/L) aqueous solution at room temperature under constant stirring. To prevent photodegradation, all experiments were conducted in the dark. Aliquots (1 mL) were withdrawn at fixed intervals (15–360 min), filtered through 0.45 μm PTFE membranes, and analyzed with a UV–vis spectrophotometer (UV1900i, Shimadzu) at 330 nm. Calibration curves were prepared in the range of 1–200 mg/L. For comparison, adsorption tests were also performed using both the sole hydrogel, LS-pAAm-DAC, and the pristine MIL-100­(Fe) (0.5 g) under identical conditions, with the same sampling schedule and the same analytical procedure. The instantaneous capacity and its equilibrium value were obtained from the determined concentrations according to eqs 3 and 4, respectively:

3 $$q_{t}\left(\mathrm{mg}/g\right)=\frac{\left(C_{0}-C_{t}\right)V}{M_{0}}$$

4 $$q_{e}\left(\mathrm{mg}/g\right)=\frac{\left(C_{0}-C_{e}\right)V}{M_{0}}$$

where *C*_0_, *C_t_*, and *C_e_* (mg/L) are the initial, time *t*, and equilibrium concentrations, respectively; *V* (L) is the solution volume, and *M*_0_ (g) is the dry mass of the composite, LS-pAAm-DAC hydrogel, or MIL-100­(Fe), depending on the system considered.

After each LS-pAAm-DAC/MIL-100­(Fe) adsorption run, composite samples were regenerated by solvent-assisted desorption in a methanol/acetic acid (9:1 v/v) mixture, with the specimens immersed in 50 mL of this solution and gently mixed with an orbital shaker (150 rpm) at room temperature. Aliquots of the desorption solution were periodically collected and analyzed by UV–vis at 330 nm until the NPX-Na concentration remained constant between two consecutive sampling times, indicating complete regeneration. The adsorption-desorption procedure was repeated for five cycles.

#### 2.5.2 Photo-Fenton Degradation Experiments of NPX-Na

The photocatalytic degradation of naproxen was carried out under UVA (λ = 365 nm) irradiation using a Photolab LED275–2/365–13i lamp (Apria Systems S.L.). LS-pAAm-DAC/MIL-100­(Fe) composite (0.5 g/L) was added to 600 mL of a 100 mg/L NPX-Na solution together with 10 mM H_2_O_2_. No pH adjustment was made (initial pH = 6.8). The suspension was gently stirred at room temperature and irradiated with the UVA lamp. Aliquots were collected at fixed time intervals (0–360 min), filtered, and analyzed by UV–vis spectroscopy at 330 nm. Blank experiments (without LS-pAAm-DAC/MIL-100­(Fe)) were performed for comparison. A 10000 U/mL bovine liver catalase stock solution, corresponding to a concentration of 2.6 mg/mL, was prepared using the lyophilized powder with an activity of 3809 U/mg (solid) and 4966 U/mg (protein) and stored at 4 °C. At each predetermined sampling time during the NPX-Na degradation experiment, a 4 mL aliquot of the suspension was withdrawn and immediately spiked with 10 μL of the catalase stock solution, corresponding to 100 U, to decompose residual H_2_O_2_ and arrest photo-Fenton reactions.^30^ The sample was gently mixed to ensure rapid quenching prior to UV–vis and HPLC/MS analysis. This dose, based on the estimated hydrogen peroxide content (13 μmol per sample), ensures complete H_2_O_2_ quenching within 30 s at room temperature. Following enzyme addition, samples were immediately filtered through 0.45 μm regenerated cellulose syringe filters prior to further analysis.

An analogous protocol with slight modifications was employed to decouple adsorption from photodegradation. The suspension was first kept in the dark (lamp off) without H_2_O_2_ and monitored until NPX-Na adsorption reached equilibrium. At that point, 10 mM H_2_O_2_ was added, and UVA (365 nm) irradiation was started (*t* = 0 for photocatalysis), after which sampling, catalase quenching, filtration, and analyses proceeded exactly as in the first experiment.

#### 2.5.3 Iron Leaching Analysis *via* ICP-OES

Potential iron release from the composite during photo-Fenton operation was assessed by ICP-OES (Optima 8300, PerkinElmer). At the end of each irradiation experiment, aliquots of the treated solution were collected and analyzed directly without dilution. Emission was monitored at Fe 238.20 nm as the primary analytical line, with 259.94 nm used as a secondary line and automatic background correction. Quantification relied on an external calibration curve prepared from certified Fe standard solutions in the concentration range of 0.1–100 ppm. Instrumental and matrix blanks were run in parallel. All measurements were performed in triplicate on independent aliquots to ensure reproducibility.

#### 2.5.4 HPLC/MS Analysis

Chromatographic separations were carried out using an LCMS-2050 HPLC system (Shimadzu) equipped with a Lichrosphere 100 RP-C18 column (5 μm particle size, 150 mm × 4.6 mm i.d.) and coupled with an Electrospray Ionization source of the mass spectrometer. The injection volume was 10 μL, and elution was performed using a 1.0 mL/min flow with a mixture of solvents A (water) and B (acetonitrile). A gradient analysis was performed, where the percentage of B was increased from 20% to 80% in 10 min; then after 6 min, the mixture was restored to 20% of B for 4 min. Quantification of NPX-Na during the photodegradation experiments was performed by external calibration using a previously established calibration curve in the 2.5–100 mg/L concentration range. An Electrospray Ionization source was used in scan mode, both in positive and negative ions, with *m*/*z* value from 110 to 600, and the mass spectrometer was operated in selected ion monitoring (SIM) mode by selecting the characteristic ions of the naproxen by-products from the literature.^31^ Nitrogen was used as a nebulizer and drying gas, with a flow of 7 L/min at 200 °C for the drying gas and 1 bar for the nebulizer gas. The capillary voltage value was set to 3.0 kV.

## 3 Results and Discussion

MIL-100­(Fe) was synthesized in crystalline form by a green aqueous route that avoids the use of toxic organic solvents and fluoride, which are typically used for this framework.^32,33^ Successful synthesis under basic conditions was confirmed by the formation of a fine orange powder following air-mediated oxidation of Fe^2+^ to Fe^3+^ (Figure S1a) and by comparison of its XRD diffraction pattern with that of a commercial sample (Figure 1, blue and black lines, respectively). The characteristic reflections of the Fe–trimesate topology are distinctly resolved,^34^ confirming successful framework formation. Minor differences in the low-angle background are observed between the reference standard and the synthesized MIL-100­(Fe) patterns. These variations are attributed to background and diffuse scattering contributions, which depend on instrumental configuration and sample preparation and do not carry phase information. No additional crystalline phases are visible within the instrumental detection limits, indicating high phase purity after the synthesis and washing sequence. The ATR-FTIR spectrum of MIL-100­(Fe) powder (Figure 2, red trace) shows the characteristic vibrational features of the iron–trimesate framework (1109 and 934 cm^–1^), including an intense carboxylate-related band at 1382 cm^–1^.^35^ The aromatic out-of-plane band features at 760 and 710 cm^–1^ are also consistent with the literature.^36^ SEM image (Figure 3a) shows the morphology of the synthesized MIL-100­(Fe) powder. The material consists of aggregated clusters of submicrometric crystallites, resulting in a rough and granular surface texture.

**Figure 1:**
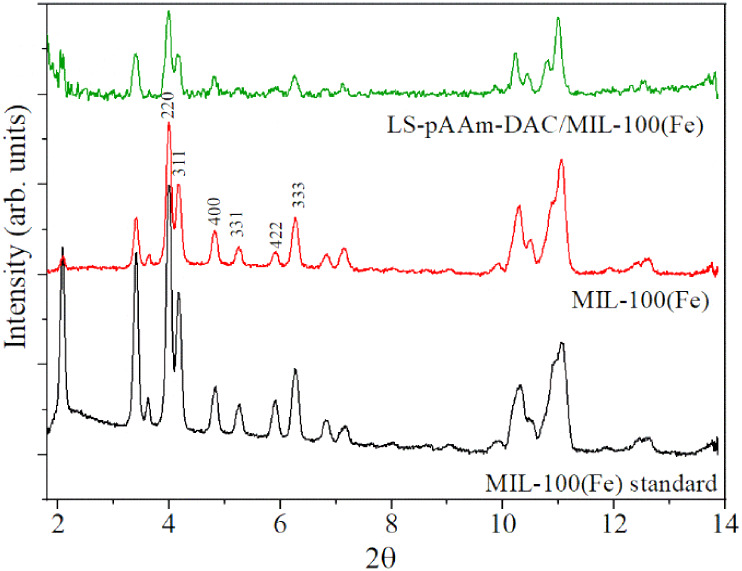
XRD diffractograms of the MIL-100­(Fe) powder from the standard reference (black line) and synthesized in this work (red line). The LS-pAAm-DAC/MIL-100­(Fe) composite is represented by the green line.

**Figure 2:**
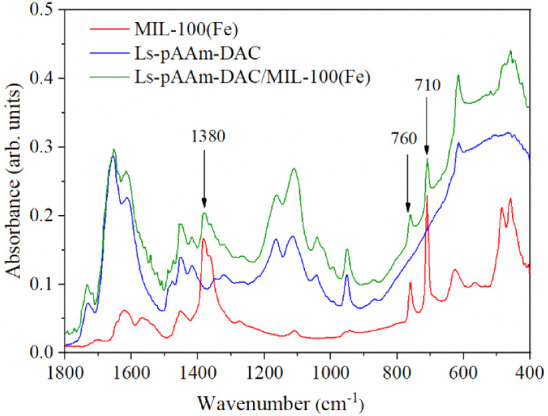
ATR-FTIR spectra of LS-pAAm-DAC, MIL-100­(Fe), and LS-pAAm-DAC/MIL-100­(Fe), showing the preservation of MIL-100­(Fe) diagnostic bands in the composite after incorporation.

**Figure 3:**
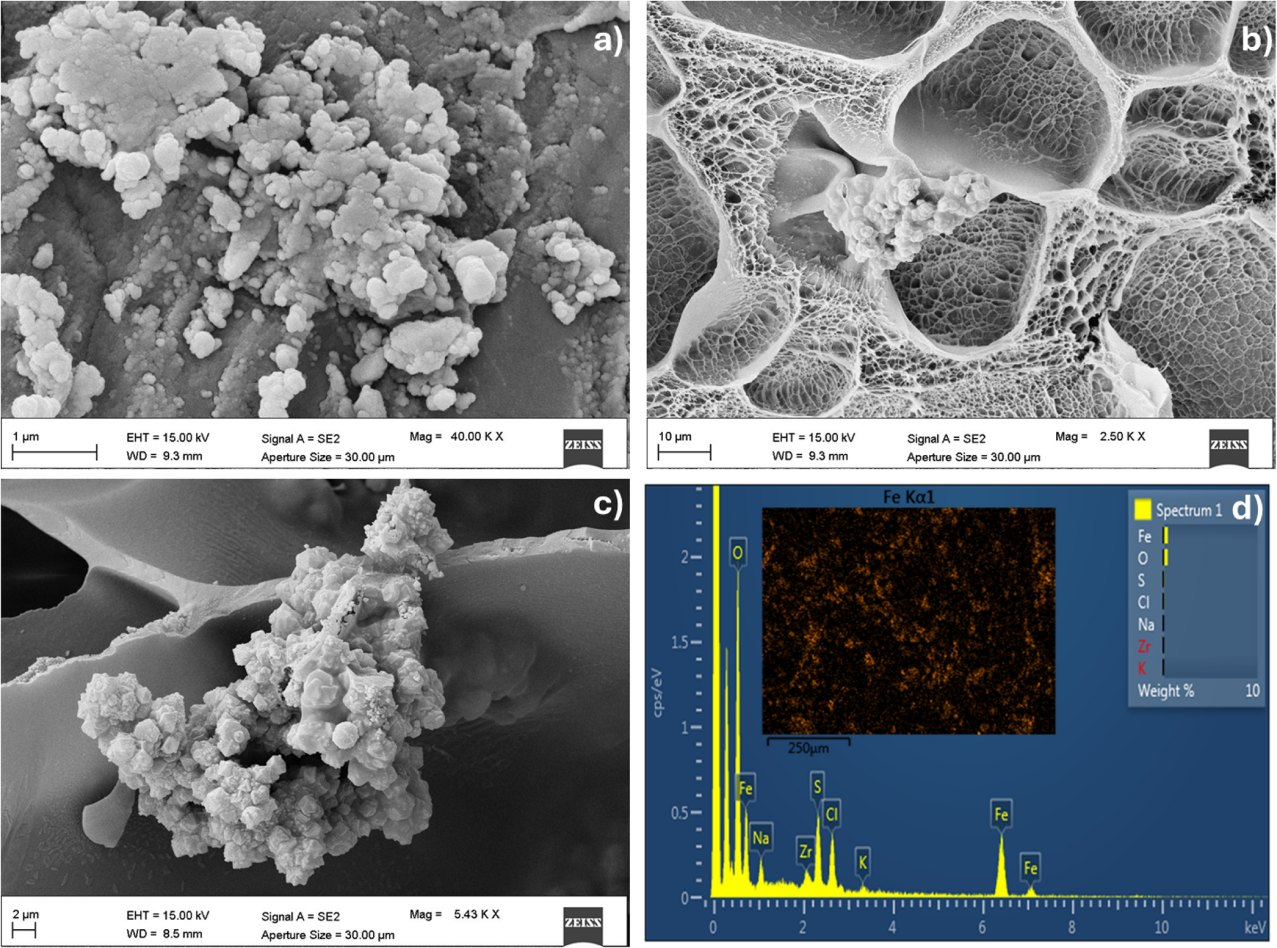
SEM and EDS characterization of MIL-100­(Fe) and LS-pAAm-DAC/MIL-100­(Fe). (a) MIL-100­(Fe) powder after synthesis and purification; (b) freeze-dried composite showing an interconnected macroporous scaffold; (c) higher-magnification detail of a particulate aggregate embedded within the polymer framework; (d) representative EDS spectrum of the composite and Fe K_α_ elemental map (inset). Scale bars: 1 μm (a), 10 μm (b), 2 μm (c).

### 3.1 Composite LS-pAAm-DAC/MIL-100­(Fe) Synthesis and Characterization

The lignin-based composite hydrogel was obtained by adding MIL-100­(Fe) to the polymerization mixture used for cationic LS-pAAm-DAC. In this way, as the radical polymerization proceeds, the framework remains immobilized within the hydrogel matrix, as suggested by the uniform orange hue of the composite (Figure S1b) without visible phase separation. XRD measurements show that the synthesized MIL-100­(Fe) pattern reproduces the positions of the characteristic Bragg reflections of the MIL-100­(Fe) standard (Figure 1, red and black traces), confirming phase identification. In the composite (Figure 1, green trace), the characteristic reflections of MIL-100­(Fe) are preserved, although with lower relative intensities. This attenuation is consistent with the low MOF loading in the composite and with differences in sample packing and diffracting volume. The persistence of MIL-100­(Fe) reflections in the composite indicates that the framework structure is maintained after the immobilization. The MOF distribution within the hydrogel is further discussed on the basis of SEM/EDS analysis.

ATR-FTIR spectra of LS-pAAm-DAC, MIL-100­(Fe), and the composite are reported in Figure 2. The hydrogel spectrum (blue trace) was taken as a reference from our previously published work on LS-pAAm-DAC.^27^ In the composite spectrum (green trace), the MIL-100­(Fe) bands are preserved and clearly overlap with the hydrogel background, providing direct spectroscopic evidence of the successful embedding and immobilization of MIL-100­(Fe) within the LS-pAAm-DAC matrix. The trimesate linker vibrations at 1109 and 934 cm^–1^ are masked by the more intense LS-pAAm-DAC signal in the same spectral region. Overall, the retention of the characteristic MIL-100­(Fe) peaks in the composite, without the emergence of additional bands indicative of framework degradation, confirms that the MOF’s chemical identity is maintained after incorporation into the polymer network.

SEM micrographs of the freeze-dried LS-pAAm-DAC/MIL-100­(Fe) composite show an interconnected microporous polymer scaffold (Figure 3b). Bright particulate domains are observed within the pores and along the internal surfaces of the polymer framework. At higher magnification (Figure 3c), these domains appear as aggregates with a grape-like morphology, consistent with MIL-100­(Fe) embedded within the hydrogel matrix.

Elemental microanalysis (EDS) was performed to support the assignment of the chemical composition of the different regions observed in the SEM micrographs. EDS spectra acquired on the continuous hydrogel scaffold are dominated by C and O, with additional contributions from S, Na, and Cl, consistent with the lignosulfonate moieties and the ionic groups/counterions of the cationic network (Figure 3d). When the electron beam is focused on the bright particulate aggregates, intense Fe K_α_ signals are detected, confirming that these domains correspond to the MIL-100­(Fe) phase. The Fe K_α_ elemental map (inset in Figure 3d) matches the location of the bright domains and shows Fe-rich regions distributed across the analyzed surface. Although Fe-rich aggregates are observed by SEM, EDS mapping confirms that MIL-100­(Fe) domains are distributed within the hydrogel scaffold. The present formulation should, therefore, be regarded as an effective immobilized composite rather than an optimized dispersion.

Prior to adsorption/desorption studies, the hydration properties of the composite were assessed to evaluate equilibrium water uptake and water retention, and the integrity of the material during the experiment was confirmed. Under the adopted conditions, the LS-pAAm-DAC/MIL-100­(Fe) composite rapidly adsorbs water during the first 240 min, reflecting the high hydrophilicity and open porosity of the LS-pAAm-DAC network (Figure 4). Thereafter, the rate decreases and approaches a plateau at 330 min, with only negligible further mass gain for the subsequent 20 h, reaching an equilibrium swelling degree of approximately 600%. The equilibrium swelling is approximately 50% lower than that of the LS-pAAm-DAC hydrogel alone,^27^ likely due to the presence of a rigid, non-swellable filler. Indeed, the crystalline MOF phase acts as a rigid physical cross-linker and topological constraint, effectively increasing the network’s cross-linking density.^37,38^ This implies a topological constraint that hinders swelling capacity and therefore reduces the available open space for water adsorption. Similar reductions in equilibrium swelling are commonly reported for nanocomposite hydrogels upon incorporation of rigid fillers into the polymer network.^39^ Differences were also observed in desorption tests compared to the LS-pAAm-DAC matrix alone.^27^ In particular, the composite exhibited rapid loss of water within the first 240 min, followed by markedly slower desorption at longer times. This behavior likely reflects a two-stage desorption process, with an initially accessible fraction released first, while a more strongly retained fraction desorbs more slowly over extended periods. The presence of MIL-100­(Fe), due to its hygroscopic character, open metal sites, and nanometer-sized porosity, contributes to stronger water–solid interactions and to a slower water release from the composite network.^40^ Within the experimental window, a small fraction of water (8%) remained trapped within the composite.

**Figure 4:**
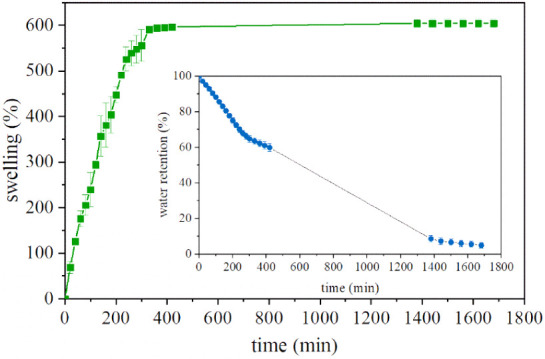
Swelling (%) behavior of the LS-pAAm-DAC/MIL-100­(Fe) composite in ultrapure H_2_O; in the inset, water retention (%) of the same sample left in open air.

### 3.2 Adsorption/Desorption of NPX-Na

The LS-pAAm-DAC/MIL-100­(Fe) composite rapidly removed NPX-Na from a 100 mg/L aqueous solution, showing a steep initial uptake followed by a slower approach to equilibrium, with an adsorption capacity *q*_*e*_ of 39 mg/g, calculated using eq 4—a value considerably higher than that of the sole LS-pAAm-DAC (ca. 21 mg/g) (Figure 5a). In addition to adsorption arising from the electrostatic interactions of NPX-Na within the cationic hydrogel network, uptake in the mesoporous cages of MIL-100­(Fe), featuring high-affinity Fe­(III) sites for carboxylates, also significantly contributes. The presence of MIL-100­(Fe) is expected to increase both the number and density of accessible binding sites,^41^ as confirmed by measurements with pristine MIL-100­(Fe), which exhibited an adsorption capacity of ca. 130 mg/g at 100 mg/L NPX-Na (Figure S2). Considering this high adsorption capacity and the ca. 10 wt % MIL-100­(Fe) loading in the composite, the observed *q*_*e*_ of 39 mg/g for LS-pAAm-DAC/MIL-100­(Fe) effectively reflects the combined contribution of both components. A correlation between hydrogel swelling and adsorption capacity is often observed, as increased swelling enhances the accessibility of internal pores and functional groups, thereby facilitating solute diffusion and promoting adsorption.^42^ Notably, in the present work, the LS-pAAm-DAC/MIL-100­(Fe) composite exhibits a higher adsorption capacity than the hydrogel alone, despite approximately 50% lower swelling. This behavior could be rationalized using hydrogel transport/uptake theory, which suggests that reduced swelling does not necessarily limit adsorption capacity when binding is governed by the density of accessible functional groups rather than by bulk water uptake.^43^ In the presence of MIL-100­(Fe), the observed lower water content increases the effective positive charge density within the hydrogel, potentially enhancing Donnan partitioning of NPX^–^ into the network.^44^ The uptake kinetics can be described as a multistep process, typical of porous materials.^45^ Initial NPX-Na uptake is dominated by external mass transfer and film diffusion from the bulk solution to the composite surface, whereas the subsequent slower regime is controlled by diffusion within the LS-pAAm-DAC/MIL-100­(Fe) microstructure and by interaction with adsorption sites in both the cationic network and the MOF phase.

**Figure 5:**
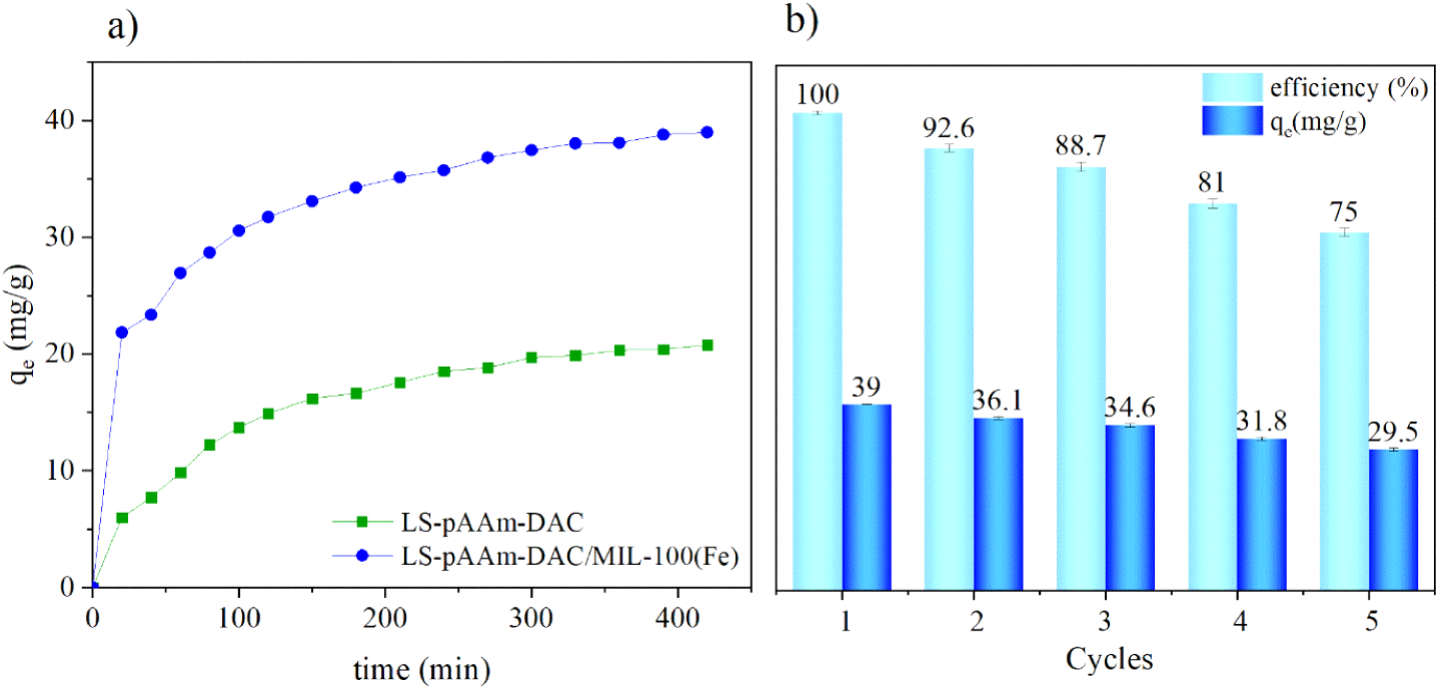
(a) Time profiles of the adsorption capacity of LS-pAAm-DAC (green line) and LS-pAAm-DAC/MIL-100­(Fe) composite (blue line) at NPX-Na (100 mg/L); (b) removal efficiency and adsorption capacity of the LS-pAAm-DAC/MIL-100­(Fe) composite over five adsorption cycles.

Reusability of the composite after NPX-Na adsorption was also evaluated by washing LS-pAAm-DAC/MIL-100­(Fe) with a methanol/acetic acid mixture to facilitate NPX-Na desorption and regeneration of the adsorption sites (Figure 5b). After five adsorption/desorption cycles, the composite remained intact with no visible cracking or fragmentation and retained much of its initial capacity, with only a modest decrease after repeated use. In Table 1, the adsorption capacity and efficiency, defined as adsorption capacity relative to the first cycle, are reported, and even after five cycles, 75% of efficiency was maintained. The small efficiency decrease likely arises from the incomplete desorption of strongly bound molecules or from partial site blocking within interfacial regions. It should also be considered that incomplete recovery of the solids after each cycle could be a plausible cause of the observed behavior.

**Table 1:** Photocatalytic Degradation of Naproxen by MIL Frameworks

MILs	Degradation Efficiency	Pseudo first-order *k* (min^–1^)	Photo-Fenton Conditions	refs
MIL-88-A	70.7%		[catalyst]: 0.25 g/L [NPX]: 50 ppm, pH: 4.0, PS: 5 mM, UVA; time: 120 min	^16^
MIL-88-A	13.0%		[catalyst]: 0.25 g/L [NPX]: 50 ppm, pH: 4.0, H_2_O_2_: 5 mM, UVA; time: 120 min	^16^
MIL-53(Al)	81.0%	0.0086	[catalyst]: 0.04 g/L [NPX]: 6 ppm, pH: 1.0, UV; time: 30 min	^48^
10%CD@MIL-88B(Fe)	90.0%	0.017	[catalyst]: 0.2 g/L [NPX]: 70 ppm, pH: 4.0, Blue LED; H_2_O_2_: 50 mM; time: 120 min	^49^
LS-pAAm-DAC/MIL-100(Fe)	90%	0.05	[catalyst]: 0.05 g/L [NPX]: 100 ppm, H_2_O_2_: 10 mM, pH: 6.4, UVA; time: 60 min	This work

### 3.3 Adsorption and Photodegradation of NPX-Na via Photo-Fenton Reaction

NPX-Na degradation was evaluated under UVA/H_2_O_2_ conditions to assess the contribution of immobilized MIL-100­(Fe) to oxidative removal beyond adsorption, by following NPX-Na UV–vis spectrum changes under photo-Fenton conditions (Figure 6). A blank control experiment was conducted under the same conditions as those for the LS-pAAm-DAC/MIL-100­(Fe) system, but without LS-pAAm-DAC/MIL-100­(Fe), and it was found that a slight degradation of NPX-Na occurred (inset in Figure 6), suggesting that the UVA/H_2_O_2_ system can induce some oxidation of the contaminant.

**Figure 6:**
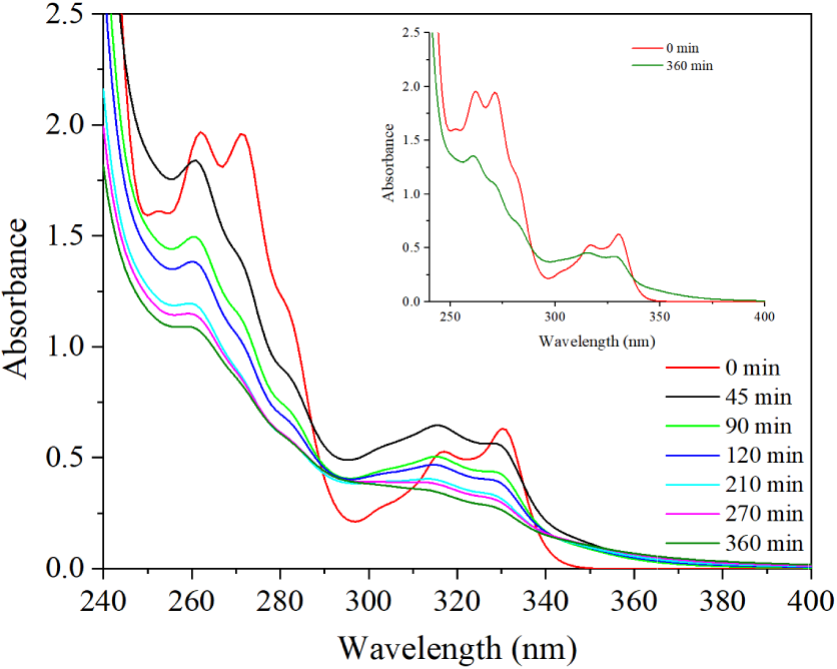
Time evolution of the UV–Vis absorption spectrum of the NPX-Na solution upon adsorption/degradation by LS-pAAm-DAC/MIL-100­(Fe) in photo-Fenton conditions. Inset: Time evolution of the UV–Vis absorption spectrum of the NPX-Na solution under UVA/H_2_O_2_ conditions.

In the presence of the LS-pAAm-DAC/MIL-100­(Fe) composite, extensive NPX-Na degradation was observed, as evidenced by significant changes in the UV–vis spectrum of the batch solution at 45 min compared to *t*_0_, likely due to the formation of degradation products, and, with time, the overall absorbance gradually decreases throughout the measurement time.

These observations are consistent with the formation of degradation products under photo-Fenton conditions and, likely, with their adsorption by the composite. Since the spectra recorded on the irradiated solutions have considerable absorbance in the 300–350 nm region, as is the case for NPX-Na, UV–vis spectroscopy alone is insufficient to conclusively assess the contribution of by-product adsorption, and measurements were repeated using HPLC/MS.

To distinguish between spectral changes due to adsorption and those due to photodegradation, the NPX-Na solution was incubated with the composite without H_2_O_2_ and with no UVA irradiation. In this dark phase, the composite absorbs NPX-Na, as shown by the decrease in absorbance in the UV–vis spectra at 330 nm (Figure S3, *t* < 0) and also confirmed by the attenuation of the NPX-Na peak at *m*/*z* 229 ([M–H]^−^) in HPLC/MS (ESI^–^). The transition to the photocatalytic stage, triggered by UVA irradiation and H_2_O_2_ addition, marked significant changes in the UV–vis NPX-Na spectrum (Figure S3, *t* > 0). For the first 45 min, a distinct increase in absorbance in the 300–340 nm region was observed, and these spectral changes are consistent with the degradation of the parent NPX-Na molecule and the simultaneous formation of photoproducts, a process that persisted over the 3 h treatment window.

Samples collected during the experiments, after catalase was added to quench the photo-Fenton reaction, were also analyzed by HPLC/MS (ESI^–^) both to follow the NPX-Na concentration decrease and to eventually identify the degradation products. As shown in Figure 7a, after the initial gradual NPX-Na decrease due to adsorption by the composite in the dark phase, a rapid NPX-Na consumption occurred when the photocatalytic stage started (*t* = 0): an NPX-Na degradation efficiency of 90% was observed after 1 h, reaching nearly 100% after 3 h.

**Figure 7:**
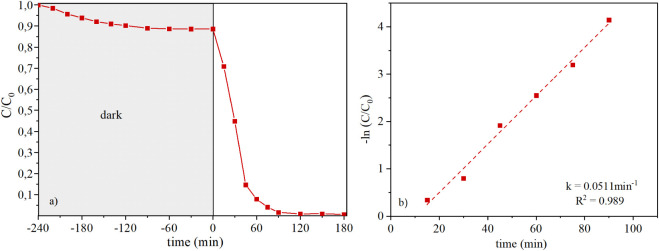
(a) Normalized concentration (*C*/*C*_0_) of NPX-Na determined by HPLC/MS during dark adsorption (*t* < 0) and photo-Fenton degradation (*t* > 0). (b) Pseudo-first-order kinetic fitting of the degradation curve.

The photodegradation kinetics were fitted using the pseudo-first-order approximation of the Langmuir–Hinshelwood model,^46,47^ which is commonly applied to heterogeneous catalytic processes, according to eq 5

5 $$-\ln\frac{C}{C_{0}}=kt$$

where *C*_0_ and *C* are the initial and the concentration at time *t* (mg/L), respectively, and *k* is the rate constant (min^–1^). From the fitting (Figure 7b), the pseudo-first-order *k* = 0.051 min^–1^ (*R* ^2^ = 0.989) was determined. This value is significantly higher than those obtained for NPX-Na degradation by other MIL-based systems (Table 1), which is particularly noteworthy considering the relatively low MIL-100­(Fe) loading (10%) within the composite. Such efficiency suggests that the hydrogel/MOF composite architecture enables effective coupling between adsorption and heterogeneous photo-Fenton degradation. In particular, the cationic hydrogel network may promote local enrichment of NPX-Na near accessible MIL-100­(Fe) domains, while the MOF provides surface-accessible Fe sites capable of activating H_2_O_2_ under UVA irradiation. The degradation process is likely mediated by reactive oxygen species generated at accessible Fe sites of MIL-100­(Fe) under UVA/H_2_O_2_ conditions.^12–14^ Based on previous studies on Fe-based MOFs and MIL-100­(Fe)-derived photo-Fenton systems, hydroxyl radicals are expected to play a major role in NPX oxidation, although the contribution of superoxide-related species cannot be excluded in the absence of radical trapping or EPR analyses.^13^ The heterogeneous photo-Fenton process is therefore expected to occur predominantly at surface-exposed or interfacial Fe sites rather than uniformly throughout^13^ the internal pore volume of the MOF. Molecules adsorbed within less accessible pore regions may contribute to pollutant uptake without necessarily participating directly in rapid oxidative degradation. Therefore, both adsorption and catalytic accessibility should not be considered equivalent in the present system. No direct evidence of modified Fe^3+^/Fe^2+^ redox cycling was obtained, and this aspect requires dedicated spectroscopic or electrochemical investigation. Beyond its kinetic performance, the ability to easily handle and recover the adsorbing/catalyst material from the aqueous phase represents a further practical advantage, addressing the common challenge of catalyst loss and secondary pollution in wastewater treatment.

As stated above, HPLC/MS was used to identify products formed in the NPX-Na photodegradation, and peak assignment was made by comparison of the collected HPLC/MS data with those reported in the literature for NPX photodegradation in water, matching the corresponding negative-ion *m*/*z* values.^31^ Different peaks were observed in the recorded chromatograms, but two signals were particularly evident and agreed with the literature reports. Both of these signals were tentatively assigned to phthalic acid derivatives (Table 2) formed during NPX-Na photodegradation, following the oxidative path typical for ketones and similar compounds: Norrish-type cleavage, oxygen addition, hydrolysis, and eliminations.^31^ The formation of highly oxidized aromatic cleavage products is consistent with ROS-mediated oxidative degradation pathways reported for Fe-based photo-Fenton systems.

**Table 2:**
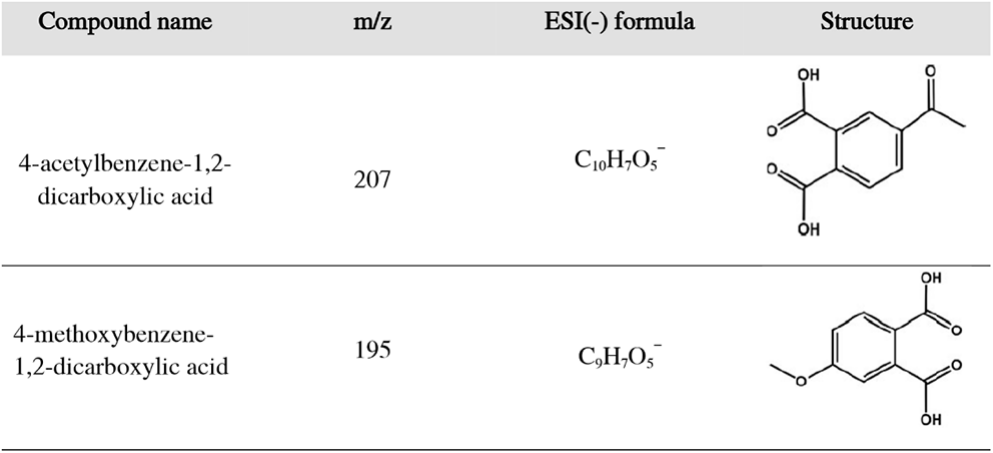
HPLC/MS Features of Naproxen Photoproducts Detected During Photo-Fenton Reaction with LS-pAAm-DAC/MIL-100­(Fe)

In particular, the two identified peaks at *m*/*z* 207 (C_10_H_7_O_5_^–^) and *m*/*z* 195 (C_9_H_7_O_5_^–^), corresponding to 4-acetylbenzene-1,2-dicarboxylic acid and 4-methoxybenzene-1,2-dicarboxylic acid, respectively, increased upon irradiation. Although only single-stage MS data were collected, the close agreement in exact *m*/*z* values with the literature data supports these tentative assignments. The pH of all collected samples remained essentially constant throughout both the dark incubation and irradiation stages and was in the 6.16–6.57 range. Under these conditions, NPX persists in its anionic form; therefore, adsorption is mainly governed by electrostatic attraction to the cationic network. ICP-OES analysis performed at the end of the experiment revealed no Fe leaching from the composite into the solution, at least within the instrumental resolution, confirming that under the applied photo-Fenton conditions, the MIL-100­(Fe) framework remained embedded within the hydrogel and structurally intact, available for repeated catalytic cycles under oxidative conditions.

## 4 Conclusions

A lignin-based cationic hydrogel embedding MIL-100­(Fe) was successfully developed as a recoverable material that integrates adsorption and heterogeneous photo-Fenton degradation capabilities. Its performance was shown in the removal of NPX-Na from water. Immobilization of the MOF within the LS-pAAm-DAC network preserves its crystallinity and catalytic functionality while overcoming the intrinsic handling and recovery limitations of powdered MIL-100­(Fe). The composite shows enhanced adsorption capacity compared with the pristine hydrogel and rapid uptake, arising from the coexistence of electrostatic binding sites in the polymer matrix and additional high-affinity adsorption domains on the MOF. Reusability tests demonstrate that the material maintains a large fraction of its initial adsorption capacity over multiple adsorption/desorption cycles, supporting its practical applicability.

Under UVA irradiation in the presence of H_2_O_2_, the pollutant is efficiently degraded through a heterogeneous photo-Fenton process, likely occurring at accessible surface/interfacial Fe sites of MIL-100­(Fe), potentially facilitated by prior adsorption and local enrichment within the composite network. The iron concentration originating from the leaching of the composite remained below the instrumental detection limit even after irradiation, confirming the structural stability of the composite and the effective confinement of the MOF within the polymer network.

By combining renewable lignin-based polymers with an iron MOF catalyst, this work provides a sustainable capture-and-convert strategy for the treatment of anionic NSAIDs. Future studies should further investigate the long-term operational stability, mechanistic understanding, and practical optimization of this hybrid catalytic platform under photo-Fenton conditions. In addition, validation in more complex water matrices and continuous flow systems will be important to further assess the environmental applicability of the proposed material.

## Supplementary Material



## Notes

Images of MIL-100­(Fe) powder and LS-pAAm-DAC/MIL-100­(Fe) composite; adsorption capacity of MIL-100­(Fe) at 100 mg/L NPX-Na; time-resolved UV–vis spectra of NPX-Na in the presence of the LS-pAAm-DAC/MIL-100­(Fe) composite during adsorption and photodegradation (PDF)
