# Supplementary material for: Lignin-Based Cationic Hydrogels Incorporating MIL-100(Fe) for Combined Adsorption and Photo-Fenton Degradation of Naproxen Sodium
Source: ACS Appl Eng Mater. 2026 Jun 5;4(6):3120–30. doi: 10.1021/acsaenm.6c00333 (PMC13317589; doi:10.1021/acsaenm.6c00333)
Supplement: Supplementary file 1 [file em6c00333_si_001.pdf]

Supporting Information

Lignin-Based Cationic Hydrogels Incorporating MIL-100(Fe) for Combined Adsorption and Photo-Fenton Degradation of Naproxen Sodium

Simone Ranieri,[1] Paola Astolfi,[1] Marco Parlapiano,[1] Massimiliano Sgroi,[1] A. Rabdel Ruiz-Salvador,[2,4] Menta Ballesteros,[3,4*] Michela Pisani[1]*

[1]Department of Science and Engineering of Materials, Environment and Urban Planning, Marche Polytechnic University, Via Brecce Bianche 12, 60131 Ancona, Italy

[2]Department of Physical, Chemical and Natural Systems Universidad Pablo de Olavide, Ctra. de Utrera, Km. 1, 41013 Seville, Spain

[3]Department of Molecular Biology and Biochemical Engineering, Experimental Sciences Faculty, Universidad Pablo de Olavide, Ctra. de Utrera km 1, 41013 Seville, Spain

[4]Center for Nanoscience and Sustainable Technologies (CNATS), Universidad Pablo de Olavide, Ctra. Utrera km. 1, 41013, Seville, Spain

*Corresponding Author: p.astolfi@staff.univpm.it

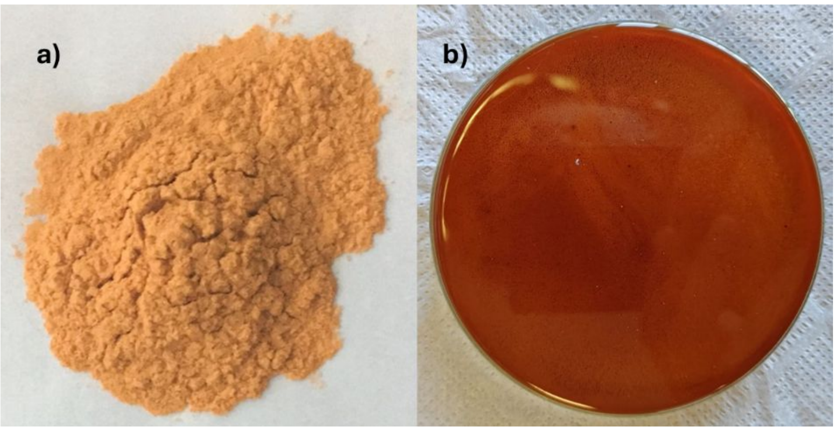

Figure S1. a) MIL-100(Fe) powder; b) LS-pAAm-DAC/MIL-100(Fe) composite.

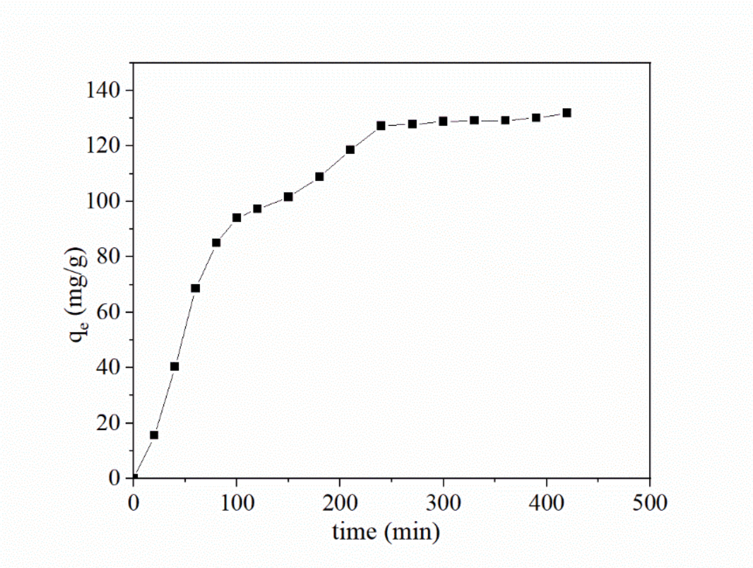

Figure S2. Adsorption capacity of MIL-100(Fe) at 100 mg/L NPX-Na.

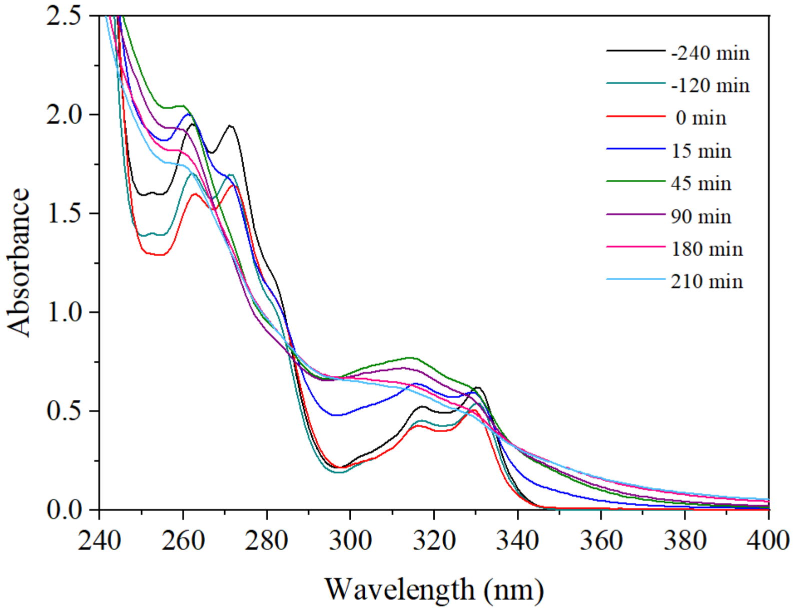

Figure S3. Time-resolved UV-Vis spectra of NPX-Na in the presence of the LS-pAAm-DAC/MIL-100(Fe) composite: dark adsorption phase (t < 0) and photocatalytic degradation phase (t > 0) upon UVA/H_2O_2 treatment
